# Factors associated with job finding anxiety among senior health sciences students: the role of attitudes toward artificial intelligence

**DOI:** 10.1186/s12909-026-08905-5

**Published:** 2026-02-28

**Authors:** Sevgi Pakiş Çetin, Nurcan Bilgin

**Affiliations:** 1 Present Address: Department of Fundamentals Nursing, Manisa Celal Bayar University, Manisa, Türkiye Turkey; 2 Department of Nursing Management, Manisa Celal Bayar University, Manisa, Türkiye Turkey

**Keywords:** Students, Anxiety, Artificial intelligence, Nursing, Midwifery, Physiotherapy and rehabilitation, Social work

## Abstract

**Background:**

Today, university students are experiencing increasing anxiety about finding a job after graduation. One of the factors intensifying this concern is the rapid advancement of artificial intelligence (AI) technologies and their potential to replace human labor in certain professions.

**Methods:**

This study examined the association between attitudes toward AI and job finding anxiety (JFA), as well as other factors related to JFA, among senior students in health-related disciplines. The study was conducted as an analytical cross-sectional design. Data were collected using the Descriptive Information Form, the General Attitude Towards Artificial Intelligence Scale, and the Job Finding Anxiety Scale. The study population consisted of 478 students, and the sample included 326 senior students in the Faculty of Health Sciences at a university between November and December 2024.

**Results:**

The analysis revealed that internet technology usage status, department, and knowledge about the use of AI in healthcare were significantly associated with students’ JFA. The multiple regression model explained 16.9% of the variance in JFA (adjusted R² = 0.169). A weak positive correlation was found between students’ internet technology use and their JFA scores (*r* = 0.168, *p* < 0.05). No significant relationship was identified between students’ attitudes toward AI and their JFA.

**Conclusions:**

The findings indicate that students’ JFA is shaped more by their academic context, use of internet technologies, and knowledge of the application of AI in healthcare than by their general attitudes toward AI. Accordingly, strategies to reduce JFA among health sciences students should prioritize the development of AI-related professional competence rather than attempting to modify general attitudes toward AI.

**Clinical trial number:**

not applicable.

**Supplementary Information:**

The online version contains supplementary material available at 10.1186/s12909-026-08905-5.

## Introduction

Nowadays, JFA among university students has emerged as a prominent psychological problem [1]. JFA is defined as individuals’ concern about being unable to secure employment and the possibility of remaining unemployed [2]. Intense competition and uncertainties in the contemporary job market, high familial and societal expectations regarding employment, financial pressures associated with independent living after graduation, and concerns about future career prospects are among the factors contributing to JFA [1]. Furthermore, the widespread prevalence of unemployment, especially among young people and university graduates, exacerbates these worries [3]. Such anxiety can lead to negative emotional states, impaired interpersonal relationships, heightened depressive symptoms, and increased suicidal ideation among university students [2]. Therefore, addressing JFA is a critical issue requiring urgent attention. According to the Organisation for Economic Co-operation and Development’s (OECD) Education at a Glance 2024 report, the unemployment rate for young adults aged 25 to 34 with tertiary education in Türkiye is 11.5%, which is nearly two and a half times higher than the OECD average of 4.7% [4]. Furthermore, the Turkish Statistical Institute’s (TUIK) Higher Education Employment Indicators 2023 report highlights that the undergraduate field with the highest employment rate is “health and welfare,” at 87.4%. Specifically, employment rates are reported as 90.9% for nursing, 92.4% for midwifery, 70.1% for social work, and 78.6% for physiotherapy and rehabilitation [5]. These data indicate that employment security is not homogeneous even among students receiving education in the health sciences.

In addition to these structural employment uncertainties, AI, as one of the main driving forces behind the transformation of the labor market, provides an important context for shaping university students’ JFA [6]. Globally, healthcare systems are confronted with challenges, including excessive workloads, insufficient funding, shortages of qualified personnel, and unforeseen stressors such as professional burnout, demographic aging, and the COVID-19 pandemic. The integration of AI into healthcare systems offers a multifaceted approach to reducing these challenges. By employing advanced computational algorithms and data-driven analytical techniques, AI can support evidence-based clinical decision-making, standardize clinical workflows, enhance the utilization of healthcare resources, and alleviate the operational burden [7, 8]. Consequently, these capabilities may contribute to improved systemic efficiency within healthcare infrastructures [8, 9].

Despite these potential benefits, concerns regarding the disruptive implications of AI on the labor market affect a broad spectrum of the population, including students [10, 11]. AI has the potential to reshape future labor dynamics, significantly influencing students’ career planning. While some students believe that AI will automate routine tasks and place greater emphasis on roles requiring creativity and critical thinking, others express concern about the potential rise in unemployment resulting from technological advancements [11].

Although studies have examined AI and JFA as separate research topics, to the best of our knowledge, no study has simultaneously investigated JFA and attitudes toward AI among health sciences students. This study aims to fill that gap by exploring the perspectives of students in health-related fields regarding AI, raising their awareness of ongoing innovative developments, and assessing their anxiety about job finding after graduation. Specifically, the study seeks to examine the association between attitudes toward AI and job-finding anxiety among senior university students in nursing, midwifery, physiotherapy and rehabilitation, and social work departments, as well as to identify factors associated with job-finding anxiety.

### Research questions

- What are the levels of JFA among health sciences students?
- What are the levels of positive and negative attitudes toward AI among health sciences students?
- Is there a relationship between health sciences students’ JFA and their positive and negative attitudes toward AI?
- Is there a significant difference in health sciences students’ JFA levels according to their sociodemographic characteristics?
- What factors are associated with JFA among health sciences students?

### Theoretical framework

The Technology Acceptance Model (TAM), developed by Davis (1989), provides a theoretical framework for explaining the role of individuals’ perceptions in shaping their attitudes toward new technologies and their subsequent usage behaviors. According to the model, perceived ease of use and perceived utility determine an individual’s attitude towards technology; this attitude, in turn, influences technology acceptance and usage behavior [12, 13]. In this context, when AI is perceived as a tool that supports individual competencies in working life, it may help students feel more prepared for their future professional roles and contribute to a reduction in perceived uncertainty regarding the job-finding process. Conversely, perceiving AI as a potential threat that may replace individuals can reduce perceived usefulness and increase feelings of uncertainty, thereby triggering JFA. Therefore, within the framework of the TAM, this study suggests that students’ attitudes toward AI may indirectly influence their JFA.

## Materials and methods

This study was planned according to the 22-item of the Strengthening the Reporting of Observational Studies in Epidemiology (STROBE) Checklist.

### Type of research

This research is analytical cross-sectional.

### Setting and sample of the study

Data were collected from senior students enrolled in the Faculty of Health Sciences at a university between 22 November and 20 December 2024. The study population consisted of 478 students, and based on the known population size, the minimum required sample size was calculated as 214 using the OpenEpi online database, with a 95% confidence interval and a 5% margin of error [14]. Although the minimum required sample size was calculated as 214 using stratified sampling based on academic departments, data collection continued until the end of the study period to maximize participation. In this context, the stratified sampling approach was used to ensure minimum representation from each department rather than to maintain proportional quotas. As participation was voluntary, a higher number of eligible students agreed to take part, resulting in a final sample of 326 students. All participants met the inclusion criteria and were therefore included in the analyses. The planned minimum sample size and the final number of participants by department were as follows: Nursing (planned: 93; actual: 123), Midwifery (planned: 48; actual: 94), Physiotherapy and Rehabilitation (planned: 38; actual: 62), and Social Work (planned: 35; actual: 47).

### Data collection tools

The following instruments were used for data collection: the Descriptive Information Form, the General Attitude Towards Artificial Intelligence Scale, and the Job Finding Anxiety Scale.

#### Descriptive information form

Developed by the researchers, this form includes 11 questions covering students’ sociodemographic characteristics and their perspectives on AI (Supplementary File 1).

#### The General Attitudes Towards Artificial Intelligence Scale (GATAIS)

The validity and reliability of the Turkish version of the GATAIS, originally developed by Schepman and Rodway (2020) [15], were established by Kaya et al. (2024) [16]. The scale is a 20-item, five-point Likert-type instrument (1 = strongly disagree, 2 = disagree, 3 = undecided, 4 = agree, 5 = strongly agree) with two sub-dimensions: Positive Attitude Towards Artificial Intelligence (PATAI; items 1–12) and Negative Attitude Towards Artificial Intelligence (NATAI; items 13–20). Responses are scored from 1 to 5, with all items in the NATAI sub-dimension reverse-scored so that higher scores in both sub-dimensions indicate more positive attitudes. Schepman and Rodway (2020) reported Cronbach’s alpha coefficients of 0.88 for PATAI and 0.83 for NATAI [15]. Kaya et al. (2024) found Cronbach’s alpha values of 0.82 for the PATAI sub-dimension and 0.84 for NATAI [16]. In the current study, the reliability coefficients were 0.90 for PATAI and 0.87 for NATAI.

#### The Job Finding Anxiety Scale (JFAS)

The JFAS, developed by Şanli et al. (2023), is a unidimensional instrument designed to measure university students’ anxiety about not finding job [2]. It consists of 10 items rated on a four-point Likert scale (1 = disagree, 2 = partly disagree, 3 = partly agree, 4 = agree). Eight items (1, 2, 3, 5, 6, 7, 9, and 10) are negatively worded, while two items (4 and 8) are positively worded and reverse scored. Total scores range from 10 to 40, with lower scores indicating lower anxiety and higher scores indicating greater anxiety about finding a job [2]. In the study by Şanli et al. (2023), the Cronbach’s alpha values of the JFAS were reported as 0.85 and 0.88 for the first and second sample groups, respectively [2]. In the present study, the scale demonstrated high internal consistency, with a Cronbach’s alpha value of 0.84.

### Data collection

Data were collected through face-to-face surveys carried out to participants. The questionnaires were completed in a structured manner, allowing participants to ask questions for clarification if needed. Participation was voluntary, and all responses were anonymized to ensure confidentiality.

### Data analysis

Statistical analyses were conducted using IBM SPSS Statistics Version 23.0 (SPSS Inc., Chicago, IL, USA) [17]. Descriptive statistics, including frequencies, percentages, and means, were calculated for the participants’ demographic characteristics. The normality of the data distribution was assessed using skewness and kurtosis values. The results were as follows: PATAI sub-dimension (Kurtosis = 0.643, Skewness = -0.220), NATAI sub-dimension (Kurtosis = 0.064, Skewness = -0.043), and JFAS (Kurtosis = -0.424, Skewness = -0.469). Values between − 1.5 and + 1.5 were considered indicative of a normal distribution [18]. Independent samples t-test, one-way analysis of variance, Mann-Whitney U test, Kruskal-Wallis test, Pearson correlation analysis, and multiple regression analysis were used for data analysis. Although the overall data distribution met the normality assumptions, non-parametric tests were applied for comparisons involving subgroups with sample sizes smaller than 30, in accordance with statistical recommendations. Regression assumptions were assessed prior to analysis. Linearity, normality of residuals, and homoscedasticity were examined and confirmed. Multicollinearity diagnostics, including variance inflation factor (VIF) and tolerance values, were within acceptable limits and are presented in Table 4. Independence of errors was assessed using the Durbin–Watson statistic. Therefore, all assumptions required for multiple regression analysis were satisfied. Additionally, internet technology use was assessed using a self-reported 11-point numeric rating scale ranging from 0 to 10, where 0 indicated no use and 10 indicated very high use. Although the scale was visually presented as a 10 cm horizontal line to facilitate participant response, scores were recorded as integer values between 0 and 10 and treated as a continuous variable in the analyses. Moreover, dummy variables are frequently employed in regression analyses to represent categorical variables with more than two categories, such as education level [19, 20]. In this study, the variables “department” and “knowing the use of AI in health” were included in the regression model as dummy variables. Correlation coefficients were interpreted based on the following criteria: 0.00–0.10 as very weak, 0.10–0.39 as weak, 0.40–0.69 as moderate, 0.70–0.89 as high, and 0.90–1.00 as very high [21]. The level of statistical significance was set at *p* < 0.05.

### Ethical considerations

This study adheres to the ethical principles outlined in the Declaration of Helsinki and received formal approval from the Health Sciences Ethics Committee of the Faculty of Medicine at Manisa Celal Bayar University (Decision Date: November 20, 2024; Decision No: 20.478.486/2719). All participants were informed about the purpose and procedures of the study and gave written informed consent before data collection. Confidentiality, voluntary participation and anonymity were meticulously maintained throughout the research process.

## Results

Of the students, 37.7% were enrolled in the Department of Nursing, and 85.9% were female. 95.1% had not received any training on AI, and 76.7% had not received training on innovation. 66.3% reported having partial knowledge of AI, and 61.3% stated they had partial knowledge about its use in the health sector. Additionally, 52.1% did not perceive AI as a threat to their profession, and 73.6% expressed partial trust in AI technology. Regarding internet usage, 33.4% reported using the internet daily for 121–180 min (Table 1).

**Table 1:** Sociodemographic Characteristics of the Students (*n* = 326)

Characteristics		*n*	%
Department	Nursing Midwifery Physiotherapy and Rehabilitation Social Work	123 94 62 47	37.7 28.8 19.0 14.5
Gender	Female Male	280 46	85.9 14.1
Receiving training on AI	Yes No	16 310	4.9 95.1
Receiving training on innovation	Yes No	76 250	23.3 76.7
Knowing the concept of AI	Yes Partly No	75 216 35	23.0 66.3 10.7
Knowing the use of AI in healthcare	Yes Partly No	53 200 73	16.3 61.3 22.4
Perceived threat of AI to the profession	Yes Partly No	27 129 170	8.3 39.6 52.1
Having trust in AI technology	Yes Partly No	48 240 38	14.7 73.6 11.7
Daily internet usage time	0–60 min 61–120 min 121–180 min 181–240 min 241 min and over	10 49 109 80 78	3.1 15.1 33.4 24.5 23.9

The mean age of the students was 22.08 ± 1.28 years. The mean score for internet technology use was 7.27 ± 2.03. Regarding attitudes towards AI, the mean score for the PATAI subscale was 3.55 ± 0.64, while the NATAI subscale had a mean score of 3.05 ± 0.76. The mean score on the JFAS was 29.38 ± 6.67. No significant relationship was found between students’ age and their JFA scores (*p* > 0.05). However, a weak positive correlation was observed between internet technology use and job finding anxiety scores (*r* = 0.168). Additionally, no significant relationships were identified between the positive and negative attitude subdimensions of the GATAIS and the JFAS (*p* > 0.05). Correlation analysis further revealed that attitudes toward artificial intelligence were not significantly associated with job finding anxiety (Table 2).

**Table 2:** Mean Scores and Correlations of GATAIS Subdimensions, JFAS, and Numerical Variables (*n* = 326)

Numerical variables and scales	Mean ± Sd	Min-Max	PATAI Subdimension	NATAI Subdimension	JFAS
1. Age	22.08±1.28	20–30	r: 0.000	r: 0.016	r: -0.067
2. Use of internet technology†	7.27±2.03	0–10	r: 0.088	r: 0.045	r: 0.168*
3. PATAI Subdimension	3.55±0.64	1.5–5	-	r: 0.001	r: 0.031
4. NATAI Subdimension	3.05±0.76	1–5	**-**	**-**	r: -0.037
5. JFAS	29.38 ± 6.67	12–40	-	-	-

r: Pearson correlation analysis *p < 0,05,†Obtained based on self-report on a 10-point scale with a single question.

PATAI: Positive Attitudes Towards Artificial Intelligence

NATAI: Negative Attitudes Towards Artificial Intelligence

JFAS: The Job Finding Anxiety Scale

A statistically significant difference was found in JFAS mean scores based on their academic department, receipt of innovation-related education, and level of knowledge about the use of AI in healthcare (*p* < 0.05). Specifically, students in the Nursing department had significantly lower job finding anxiety scores than those in the physiotherapy and rehabilitation and social work departments. Likewise, students in physiotherapy and rehabilitation reported lower scores than those in social work, and students in midwifery had lower scores than those in social work. In terms of innovation education, students who had not received education on innovation exhibited higher JFAS than those who had received such education. Similarly, students who were fully or partially informed about the use of AI in healthcare had lower job finding anxiety scores than those who reported no knowledge. No significant correlations were observed between job finding anxiety scores and students’ gender, receiving education on AI, general knowledge about AI, perception of AI as a threat to the profession, trust in AI technology, or daily internet usage time (*p* > 0.05) (Table 3).

**Table 3:** Sociodemographic Characteristics of Students and Mean Scores on the JFAS (*n* = 326)

Characteristics		Mean ± Sd	Test statistics
Department	Nursing (a) Midwifery (b) Physiotherapy and Rehabilitation(c) Social Work (d)	27.00 ± 6.26 29.11 ± 6.40 30.67 ± 7.13 34.44 ± 4.09	F: 17.324 p: 0.000* a < c< d**, b < d**
Gender	Female Male	29.58 ± 6.69 28.17 ± 6.54	t: 1.327 p: 0.185
Receiving training on AI	Yes No	27.06 ± 6.13 29.50 ± 6.69	z: -1.487 p: 0.137
Receiving training on innovation	Yes No	27.22 ± 6.18 30.04 ± 6.69	t: -3.267 p: 0.001*
Knowing the concept of AI	Yes Partly No	27.81 ± 6.97 29.75 ± 6.58 30.45 ± 6.20	F: 2.892 p: 0.057
Knowing the use of AI in healthcare	Yes (a) Partly (b) No (c)	27.84 ± 7.80 28.81 ± 6.33 32.05 ± 6.03	F: 8.323 p: 0.000* a = b < c**
Perceived threat of AI to the profession	Yes Partly No	29.96 ± 6.41 30.00 ± 6.13 28.81 ± 7.08	KW: 2.004 p: 0.367
Having trust in AI technology	Yes Partly No	29.00 ± 5.99 29.57 ± 6.85 28.65 ± 6.41	F: 0.401 p: 0.670
Daily internet usage time	0–60 min 61–120 min 121–180 min 181–240 min 241 min and over	23.90 ± 6.67 30.02 ± 6.84 29.03 ± 6.61 30.55 ± 6.50 28.97 ± 6.57	KW: 8.938 p: 0.063

*p < 0.05, **Post hoc Tukey’s-b test result, t: t test in independent groups, F: One-way analysis of variance, z: Mann Whitney U test, KW: Kruskal Wallis test

Internet technology usage, academic department, and knowledge regarding the use of AI in healthcare were found to be significantly associated with JFA in the multiple regression model (F = 14.177, *p* < 0.001). However, the standardized regression coefficients indicated small effect sizes. Specifically, internet technology usage showed a weak effect on JFA (β = 0.141), suggesting a limited practical impact despite statistical significance. Similarly, academic department (β = 0.192) and knowledge about the use of AI in healthcare (β = 0.160) also demonstrated small effect sizes. Overall, the regression model explained 16.9% of the variance in job finding anxiety (adjusted R² = 0.169), indicating that although the model was statistically significant, job finding anxiety is likely influenced by additional academic, contextual, and psychosocial factors not included in the model (Table 4).

**Table 4:** Factors Associated with the Total Score of the JFAS: Multiple Regression Analysis Results (*n* = 326)

Multiple regression model	B	S.H.	β	t	*p*	95% Confidence Interval for B	Collinearity Statistics		
							Tolerance		VIF
(Constant)	23.573	1.394		16.908	0.000	20.830 26.316			
Use of internet technology	0.462	0.166	0.141	2.774	0.006	0.134 0.789	0.992	1.008	
Department (0. Nursing/Midwifery, (1) Physiotherapy and Rehabilitation, (2) Social Work)	1.747	0.791	0.192	2.209	0.028	0.191 3.303	0.338	2.957	
Department (0. Nursing, (1) Midwifery/Physiotherapy and Rehabilitation, (2) Social Work)	1.614	0.966	0.165	1.671	0.096	-0.286 3.515	0.261	3.833	
Receiving training on innovation (0: Yes, 1: No)	-0.257	0.993	-0.016	-0.259	0.796	-2.211 1.698	0.645	1.551	
Having knowledge about the use of AI in healthcare (0: Yes/Partially, 1: No)	2.557	0.851	0.160	3.006	0.003	0.883 4.231	0.904	1.106	

R: 0.426 Adjusted R^2^: 0.169 F: 14.177 p: 0.000 Durbin-Watson: 1.985

## Discussion

Unemployment among university students extends beyond a mere economic concern and constitutes a significant public health issue due to its adverse effects on psychological well-being, particularly unemployment-related anxiety [22]. In this context, the present study examined the impact of attitudes toward AI on JFA among senior students enrolled in health-related disciplines (nursing, midwifery, physiotherapy and rehabilitation, and social work). The primary finding of this study is that attitudes toward AI—both positive and negative—are not significantly associated with JFA among participants. This finding directly addresses the main research question and suggests that employment-related concerns may not be directly influenced by individuals’ attitudes toward AI.

The findings revealed that students in health-related programs exhibited above-average levels of job finding anxiety, with a mean score of 29.38 out of a possible 40. This aligns with previous research indicating high levels of job finding anxiety among university students [23, 24], although other studies have reported moderate levels of anxiety [3]. The high anxiety observed in this study may be attributed to the competitive nature of the current job market and the uncertainty surrounding future employment prospects [25]. Moreover, the structural challenges of the labor market in Türkiye exacerbate this issue. Specifically, the unemployment rate among university graduates in Türkiye is nearly two and a half times higher than the OECD average of 4.7%, underscoring the significant barriers to labor market participation faced by graduates. This issue may be attributed to the weak alignment between higher education outcomes and labor market demands in Türkiye.

In the present study, students demonstrated a generally positive attitude toward AI (with a mean score of 3.55 out of 5). This finding is consistent with previous research indicating that students in health-related disciplines tend to hold favorable views toward AI [6, 26, 27]. Similarly, a systematic review by Mousavi Baigi et al. (2023) reported that 76% of the included studies found health sciences students to have a positive and optimistic attitude regarding the clinical and future applications of AI [28]. Supporting this, Amiri et al. (2024), in their systematic review and meta-analysis, noted that 65% of students expressed positive attitudes toward AI. These findings suggest a growing acceptance of AI technologies within the healthcare sector [29].

Contrary to expectations, the findings of the study revealed no significant correlation between students’ mean scores on the JFAS and the sub-dimension scores of the GATAIS. This finding suggests that students’ attitudes toward AI do not have a direct and determining effect on JFA. This suggests that JFA may be more strongly shaped by structural and contextual factors—such as uncertainties in the labor market, limited employment opportunities, and economic conditions—rather than by individual attitudes toward AI. Indeed, within the framework of the TAM, individuals’ attitudes toward technology are primarily effective in explaining usage intentions and behaviors [12]; however, the model may be limited in explaining structural and economic processes, such as macro-level employment-related anxieties. In contrast to the present study, Li et al. reported a significant negative association between attitudes toward AI and JFA among undergraduate, master’s, and doctoral students [6]. While there is a lack of directly comparable studies in literature, a limited number of studies have explored the relationship between anxiety related to AI and unemployment. For instance, Uçar et al. (2024) found that university students reported high levels of anxiety regarding both AI and unemployment, with a moderate positive correlation observed between AI-related anxiety and unemployment anxiety [24]. In contrast, studies focusing specifically on students in the health sciences have reported concerns that the increasing implementation of AI in healthcare may reduce the future demand for health professionals, thereby contributing to high JFA [30–33].

In this study, a weak positive correlation was identified between students’ use of internet technology and the JFAS, indicating that greater use of internet technology was associated with higher anxiety about finding employment. Furthermore, greater use of internet technology was found to be significantly associated with higher levels of job finding anxiety. This finding suggests that students with broader access to information via internet technologies may experience increased anxiety. Although the accuracy and quality of the information students access online remain unclear, it is plausible that regularly monitoring employment opportunities, particularly among senior students nearing graduation, could contribute to high levels of anxiety. Supporting this interpretation, Uçar et al. (2024) reported that students with greater reliance on technology demonstrated higher levels of unemployment-related anxiety [24]. Similarly, Doğan et al. (2023) found that students in sports sciences who used the internet for 241 min or more per day reported significantly higher levels of JFA [11].

In this study, although a statistically significant difference was found between students’ innovation-oriented education status and their mean scores on the JFAS in bivariate analyses, this association lost its significance in the regression analysis. This finding may indicate that the effect of innovation-oriented education on JFA diminishes when considered alongside other individual and contextual factors, rather than acting as an independent variable.

In this study, academic department and having knowledge about the use of AI in healthcare were identified as factors significantly associated with JFA. According to the TUIK (2023), the employment rates for recent graduates were reported as 90.9% for nursing, 92.4% for midwifery, 70.1% for social work, and 78.6% for physiotherapy and rehabilitation [5]. Given these employment rates, it is expected that students enrolled in physiotherapy and rehabilitation, as well as social work programs, will exhibit higher levels of JFA than their peers in other departments. The relatively lower employment rates in these fields in Türkiye may be attributed to factors such as an oversupply of graduates and the limited availability of professional practice settings within these disciplines. The findings of this study revealed that students who lacked knowledge about the use of AI in healthcare reported higher levels of JFA. This may be attributed to the fact that students who are informed about AI applications in health are more aware of ongoing innovations in the field and are optimistic that these advancements could enhance their employment prospects. Supporting this view, Cho and Seo (2024) found that students who received AI training exhibited higher levels of AI perception, lower levels of AI-related anxiety, and more favorable attitudes toward the adoption of AI than those without such training [34]. Similarly, Al-Qerem et al. (2023) reported that students’ complex attitudes toward AI may contribute to concerns such as fear of job loss [35]. Furthermore, a qualitative study by Güzel et al. (2022) identified several perceived advantages of AI in healthcare, including reduced workload, decreased medical errors, and improvements in time-efficiency and labor savings [36]. Therefore, the findings of this study are consistent with those reported in the existing literature.

This study, conducted within the framework of the TAM, provides a useful theoretical foundation for understanding individuals’ attitudes toward AI; however, its explanatory power is largely limited to technology acceptance, perceived usefulness, and behavioural intention. In contrast, JFA is a multidimensional and macro-level phenomenon shaped by psychosocial processes, academic factors, and structural conditions, and cannot be explained solely by individual attitudes. The limited explained variance observed in the regression analysis (16.9%), together with the small effect sizes, supports the view that JFA among health sciences students reflects a complex and multifactorial structure that extends beyond technology-related characteristics. Moreover, the General Attitude Towards Artificial Intelligence Scale used in this study does not directly capture health field–specific perceptions, which may have partially limited the explanatory capacity of the model. Indeed, the literature includes studies indicating that psychological distress experienced by university students during the job-finding process is influenced not only by individual attitudes but also by psychosocial factors such as levels of social support and the use of individual strengths [23, 37].

### Limitations

To the best of our knowledge, this study addresses a relatively underexplored area by examining the relationship between health sciences students’ attitudes toward AI and JFA, thereby contributing to the existing literature. However, the study has several limitations. First, the limited availability of relevant literature constrained the depth and scope of the discussion. Second, the use of the GATAIS, which assesses general attitudes toward AI, may not have adequately captured concerns and expectations related to AI applications specific to the health field. In addition, given the multidimensional nature of JFA, the explanatory power of the proposed model remained limited. Moreover, the cross-sectional design of the study precludes causal inferences, as all variables were measured at a single point in time. Finally, because the study was conducted with students from the Faculty of Health Sciences at a single university and was based solely on participants’ self-reported responses to the measurement scales, the findings are sample-specific. Voluntary participation and the unequal distribution of participants across departments may have contributed to selection bias. Therefore, the results cannot be generalized to broader populations.

## Conclusion and recommendations

The study identified “use of internet technology,” “department,” and “having knowledge about the use of AI in healthcare” as factors significantly associated with JFA. Based on these results, several recommendations may be suggested to help reduce JFA among health sciences students.

These include providing students with realistic information about labor market conditions during department selection, supporting students in regulating their internet use to avoid excessive time spent on job-searching platforms that may increase anxiety, and strengthening institutional-level career counseling services. Additionally, future studies should replicate these findings using larger sample sizes across diverse university settings to enhance generalizability.

## Supplementary Information

Supplementary Material 1.

## Data Availability

The datasets used and/or analysed during the current study are available from the corresponding author on reasonable request.
