# Supplementary material for: Factors associated with job finding anxiety among senior health sciences students: the role of attitudes toward artificial intelligence
Source: BMC Med Educ. 2026 Feb 28;26:562. doi: 10.1186/s12909-026-08905-5 (PMC13059450; doi:10.1186/s12909-026-08905-5)
Supplement: Supplementary file 1 — Supplementary Material 1. [file 12909_2026_8905_MOESM1_ESM.docx]

**DESCRIPTIVE INFORMATION FORM**

1. **Your Department:**
   a) Nursing
   b) Midwifery
   c) Physiotherapy and Rehabilitation
   d) Social Work
2. **Your Age:** …………
3. **Your Gender:**
   a) Female
   b) Male
4. **Have you received training related to artificial intelligence?**
   a) Yes
   b) No
5. **Have you received training related to innovation?**
   a) Yes
   b) No
6. **Do you think you have knowledge about the concept of artificial intelligence?**
   a) Yes
   b) Partly
   c) No
7. **Do you think you have knowledge about the use of artificial intelligence in healthcare?**
   a) Yes
   b) Partly
   c) No
8. **Do you consider artificial intelligence technology as a threat to your profession?**
   a) Yes
   b) Partly
   c) No
9. **Do you trust artificial intelligence technology?**
   a) Yes
   b) Partly
   c) No
10. **Please indicate your level of internet technology use:** ………
    *(0 = I do not use it at all, 10 = I actively follow and use it)*
11. **Please indicate your daily internet usage time:**
    a) 0–60 minutes
    b) 61–120 minutes
    c) 121–180 minutes
    d) 181–240 minutes
    e) 241 minutes or over
